# Emergence of Fluoxetine-Resistant Variants during Treatment of Human Pancreatic Cell Cultures Persistently Infected with Coxsackievirus B4

**DOI:** 10.3390/v11060486

**Published:** 2019-05-28

**Authors:** Enagnon Kazali Alidjinou, Antoine Bertin, Famara Sane, Delphine Caloone, Ilka Engelmann, Didier Hober

**Affiliations:** Faculté de médecine, Université Lille, CHU Lille, Laboratoire de Virologie EA3610, F-59000 Lille, France; enagnonkazali.alidjinou@chru-lille.fr (E.K.A.); antoine.bertin@gmail.com (A.B.); famara.sane@chru-lille.fr (F.S.); delphine.lobert@univ-lille.fr (D.C.); ilka.engelmann@chru-lille.fr (I.E.)

**Keywords:** enteroviruses, coxsackievirus B4, persistent infection, fluoxetine, resistance, mutations

## Abstract

This study reports the antiviral activity of the drug fluoxetine against some enteroviruses (EV). We had previously established a model of persistent coxsackievirus B4 (CVB4) infection in pancreatic cell cultures and demonstrated that fluoxetine could clear the virus from these cultures. We further report the emergence of resistant variants during the treatment with fluoxetine in this model. Four independent persistent CVB4 infections in Panc-1 cells were treated with fluoxetine. The resistance to fluoxetine was investigated in an acute infection model. The 2C region, the putative target of fluoxetine antiviral activity, was sequenced. However, Fluoxetine treatment failed to clear CVB4 in two persistent infections. The resistance to fluoxetine was later confirmed in HEp-2 cells. The decrease in viral titer was significantly lower when cells were inoculated with the virus obtained from persistently infected cultures treated with fluoxetine than those from susceptible mock-treated cultures (0.6 log TCID50/mL versus 4.2 log TCID50/mL, *p* < 0.0001). Some previously described mutations and additional ones within the 2C protein were found in the fluoxetine-resistant isolates. The model of persistent infection is an interesting tool for assessing the emergence of variants resistant to anti-EV molecules. The resistance of EV strains to fluoxetine and its mechanisms require further investigation.

## 1. Introduction

The genus *Enterovirus* (*Picornaviridae* family) is a large group of small non-enveloped RNA viruses that are involved in several mild or severe acute clinical infections in humans ranging from enteric or respiratory infections, hand-foot-and-mouth disease, or conjunctivitis to acute flaccid paralysis, viral myocarditis, fulminant pancreatitis, or aseptic meningitis [1,2,3]. Some of these viruses in this group, especially type B coxsackieviruses (CVB) are also known to play a role in the development of chronic diseases, such as chronic myocarditis or type 1 diabetes [4,5,6]. Enteroviruses (EV) are well known as cytolytic viruses, but they can also establish persistent infections in vitro and in vivo, a mechanism potentially involved in the pathogenesis of chronic diseases [7].

Despite several attempts of library screening and other than a few compounds under investigation, to date no antiviral molecule has been licensed worldwide for the treatment of enteroviral infections that can sometimes be potentially life threatening to humans [8,9].

Fluoxetine is a selective serotonin reuptake inhibitor (SSRI) used for the treatment of depression or other mental disorders. This drug has been reported to display a significant antiviral activity against enteroviruses in vitro, especially *Enterovirus B* and *D* species [10,11]. The putative target of fluoxetine is the nonstructural viral protein 2C, a highly conserved region among enteroviruses. Other well-known enterovirus replication inhibitors such as, guanidine hydrochloride (GuHCl) or TBZE-029 also target 2C protein, even though the mechanism might be different. Some 2C CVB3 resistant mutants have been described with cross-resistance to all these compounds [8,10].

A model of persistent coxsackievirus B4 (CVB4) infection in pancreatic cells was established by our team and represents an interesting tool to study the activity of anti-enteroviral candidate agents, and subsequently the emergence of viral resistance to these molecules. It was previously shown that the treatment with fluoxetine can cure pancreatic cell cultures persistently infected with CVB4 [12].

We further report the emergence of resistant CVB4 variants during the fluoxetine-treatment of human pancreatic cell cultures persistently infected with the virus.

## 2. Materials and Methods

### 2.1. Cells and Reagents

HEp-2 cells (BioWhittaker, Walkersville, MD, USA) were grown in minimum essential medium (MEM) supplemented with 10% of fetal calf serum (FCS), 1% of L-glutamine, 1% of nonessential amino acids, and 1% of penicillin and streptomycin. The human ductal cell line Panc-1 (ATCC) was cultured in Dulbecco’s modified Eagle’s medium (DMEM) supplemented with 10% of FCS, 1% of L-glutamine, and 1% of penicillin and streptomycin.

Fluoxetine chlorhydrate (Lilly France, Fegersheim, France) was dissolved in dimethyl sulfoxide (DMSO) at a final concentration of 5.48 uM and was used in all experiments, as previously reported [12]. Guanidine hydrochloride (GuHCl) was purchased from Sigma-Aldrich (Saint-Quentin-Fallavier, France) and was used at a final concentration of 2 mM.

### 2.2. Virus and Persistent Infection

The diabetogenic CVB4 E2 strain, provided initially by Ji-Won Yoon (Julia McFarlane Diabetes Research Center, Calgary, Alberta, Canada), was propagated in HEp-2 cells and used to establish CVB4 persistent infections.

The model of persistent CVB4 infection of Panc-1 cells has been previously described [13,14]. Briefly, a 25 cm^2^ Nunc cell culture flask (Thermofisher Scientific, Villebon, France) containing an average of 10^6^ cells in DMEM was inoculated with CVB4 at a multiplicity of infection (MOI) of 0.01. During the acute lytic infection, the culture medium was regularly changed, and finally a stable equilibrium was found between the viral replication and cell proliferation. The medium was changed twice a week, and cells were scraped and subcultured once a week. The supernatants were collected at different time points (1, 10, 20, 21, 24, 28, and 30 weeks post infection) during the persistent infection.

### 2.3. Antiviral Activity Testing

The antiviral activity of fluoxetine was evaluated using HEp-2 cells. Cells were seeded in a 96-well cell culture plate at 1.25 × 10^4^ cells per well. Cells were inoculated with the virus at a MOI of 0.01, mixed with fluoxetine or DMSO. The plates were incubated at 37 °C, and the cell cultures were observed every day. The supernatants were collected when 100% cytopathic effect (CPE) was observed in DMSO-treated wells.

### 2.4. Determination of Viral Titer

The viral titer obtained in supernatants was assessed using the end-point dilution assay, particularly the Spearman–Karber statistical method was used to determine the tissue culture 50% infectious dose (TCID50).

### 2.5. Viral Genome Sequencing

Viral genome was sequenced using the Sanger (population) method. Viral RNA was isolated from 140 µL of viral suspension with the QIAamp Viral RNA Mini Kit (Qiagen, Courtaboeuf, France) following the manufacturer’s instructions. The whole 2C region (from nt 4039 to nt 5025) was amplified using the Superscript III One-step reverse transcription-polymerase chain reaction (RT-PCR) system with Platinum Taq DNA polymerase kit (Thermo Fischer Scientific). The amplicons were checked on a 2% agarose gel, purified on Nucleoseq columns (Machery-Nagel, Hœrdt, France), and sequenced using the BigDye Terminator v3.1 Cycle Sequencing Kit (Thermo Fischer Scientific, Les Ulis, France). The sequences were purified with the BigDye XTerminator Purification Kit. Sequenced products were analyzed on a 3500Dx genetic analyzer (Thermo Fischer Scientific). Electrophoregrams were manually edited with Seqscape software v3 (Thermo Fischer Scientific). The sequences of primers are shown in Table 1.

### 2.6. Statistical Analysis

Data were presented as mean ± SD. Graphs and analyses were performed with GraphPad Prism^®^ V6.0 software. Comparisons were performed with the Mann–Whitney *U* test with the significance set at 0.05.

## 3. Results

### 3.1. Persistent CVB4 Infection and Fluoxetine Treatment

**Figure 1:**
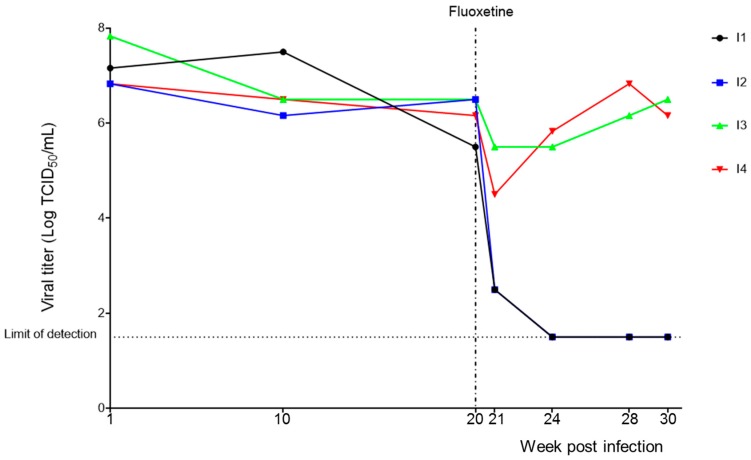
Persistent coxsackievirus B4 (CVB4) infection in Panc-1 cells and treatment with fluoxetine. Four independent persistent CVB4 infections (I1, I2, I3, and I4) were established in Panc-1 cells. At 20 weeks post infection, cells were treated with fluoxetine at 5.48 μM twice a week. The culture supernatants were collected all along the follow-up. Viral titers in supernatants were determined using the end-point dilution assay.

Four independent cultures persistently infected with CVB4 (I1, I2, I3, and I4) were established in Panc-1 cells. Culture supernatants were periodically collected and the presence of infectious particles was checked using HEp-2 cells. The viral titers obtained in the supernatants ranged from 6.83 to 7.83 log TCID_50_/mL at one week postinoculation (p.i.), 6.25 to 7.50 log TCID_50_/mL at ten weeks p.i., and 5.50 to 6.50 log TCID_50_/mL at twenty weeks p.i. (see Figure 1).

Starting from week 20 p.i., persistently infected cultures were treated twice a week with fluoxetine at 5.48 μM or DMSO, and supernatants were collected periodically to monitor the infection. The levels of infectious viral particles in persistently infected cultures I1 and I2 decreased significantly at one week of treatment (o.t.) i.e., 2.5 log TCID_50_/mL, and the virus was undetectable at four weeks posttreatment. In contrast, for persistently infected cultures I3 and I4, only a slight decrease was observed in viral titers at one week o.t., followed by a rise from the fourth week o.t. The viral titers obtained after 10 weeks of fluoxetine-treatment were still at 6.5 and 6.25 TCID_50_/mL for I3 and I4, respectively (see Figure 1). No significant changes in viral titers were observed in DMSO-treated cultures.

### 3.2. Investigation of Resistance to Fluoxetine Treatment

**Figure 2:**
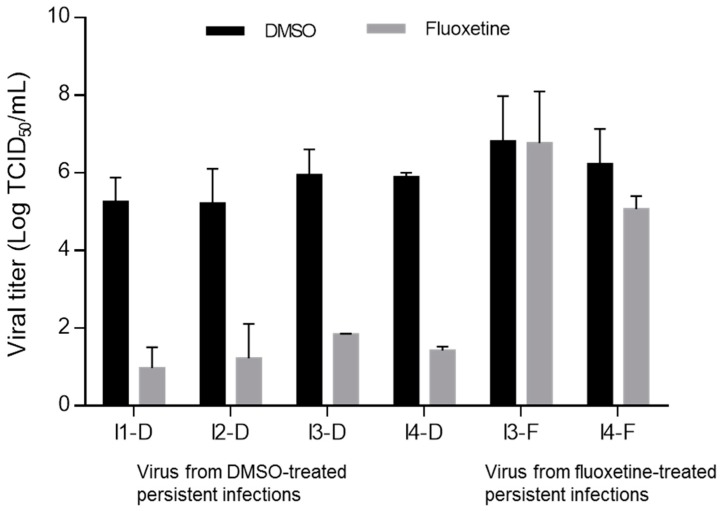
Susceptibility to fluoxetine of virus isolates obtained from treated persistently CVB4-infected cultures. Virus suspensions were collected from persistent CVB4 infections treated with DMSO (I1-D, I2-D, I3-D, and I4-D), or with fluoxetine (I3-F and I4-F) at week 8 of treatment. HEp-2 cells were inoculated with various virus suspensions in the presence of fluoxetine or dimethyl sulfoxide (DMSO). Viral titers were determined 3 days postinoculation. Data are mean ± SD of two independent experiments.

To confirm that I3 and I4 were resistant to the treatment, the susceptibility of viral suspensions to fluoxetine was evaluated in a model of acute infection. Various viral suspensions from persistently infected cultures treated with fluoxetine at 5.48 μM or DMSO were collected at week 8 o.t. They were inoculated into HEp-2 cells in the presence of fluoxetine or DMSO. Supernatants were collected on day 3 p.i., and the viral titers were determined. Figure 2 presents the extent of decrease in viral titer in fluoxetine-treated wells, as compared to DMSO-treated ones.

Virus isolates emerging from the four persistently infected cultures treated with DMSO for eight weeks (I1-D, I2-D, I3-D, and I4-D) remained highly susceptible to fluoxetine. Indeed, a mean decrease in viral titer ranging between 4 and 4.5 log TCID50/mL was observed when the isolates were inoculated into HEp-2 cell cultures in the presence of fluoxetine, as compared to the cultures inoculated with virus isolates in the presence of DMSO.

Regarding the virus obtained from persistently infected cultures treated with fluoxetine for 8 weeks, the antiviral activity of fluoxetine was strongly reduced. The mean decrease in viral titer was as low as 0.1 and 1.2 log TCID50/mL for I3-F and I4-F, respectively (see Figure 2).

No viral titer was obtained in persistently infected cultures I1 and I2 treated with fluoxetine (I1-F and I2-F) after 4 weeks of treatment; therefore, they were not tested.

Overall, the mean decrease in viral titer was significantly lower when cells were inoculated with the virus isolated from persistently infected cultures treated with fluoxetine than those from persistently infected cultures treated with DMSO (0.6 log TCID50/mL versus 4.2 log TCID50/mL, *p* < 0.0001).

The same experiments were run using GuHCl instead of fluoxetine. When the viral suspensions were inoculated into HEp-2 cells in presence of GuHCl, a mean decrease of 4.7 log TCID50/mL and 5 log TCID50/mL was observed when cells were inoculated with the virus obtained from persistently infected cultures treated with DMSO and persistently infected cultures I3-F and I4-F treated with fluoxetine, respectively for 8 weeks. There is no statistical difference between these viral titer reductions.

### 3.3. Mutations in 2C Protein Associated with Resistance to Fluoxetine

Since the 2C viral protein was reported as the target of fluoxetine antiviral activity, we investigated whether the resistance was associated with mutant variants. The sequence of the 2C region was determined in the stock virus, virus obtained from persistently infected cultures before treatment, and virus collected from persistently infected cultures treated with fluoxetine or DMSO treated for 4 and 10 weeks.

All the positions with amino-acid substitution in the different sequences as compared to CVB4 E2 reference strain (NCBI, accession: AF311939.1) are presented in Table 2.

**Figure 3:**
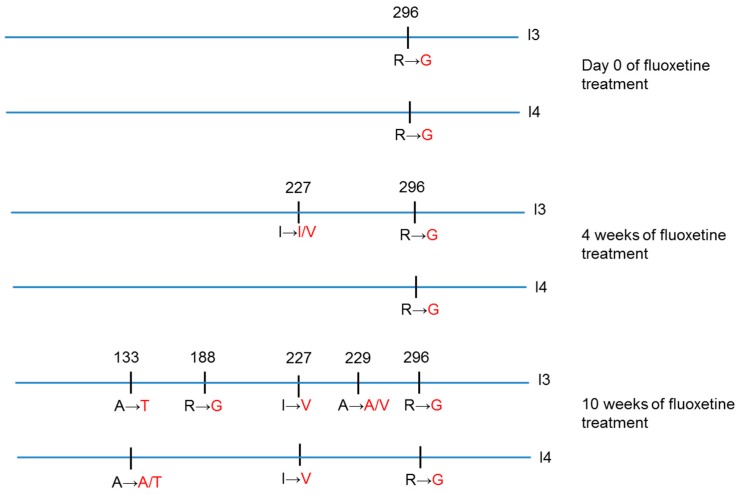
Amino-acid substitutions in fluoxetine-resistant virus. Virus suspensions were collected from persistent CVB4 E2-infected cultures at baseline, and after 4 and 10 weeks of treatment with DMSO or fluoxetine. The sequence of the whole CVB4 E2 2C region (from nt 4039 to nt 5025, 329 aa) was determined using Sanger method. The amino-acid substitutions in the sequences of fluoxetine-resistant viruses (I3 and I4) are shown.

Figure 3 focuses on mutations that appeared in the sequences of virus obtained from the fluoxetine-resistant persistently infected cultures I3-F and I4-F.

Before fluoxetine treatment, the R296G mutation was observed in I3 and I4, but not in I1, I2, and the initial stock virus. This substitution was present during all follow-ups in the sequences from fluoxetine or DMSO-treated I3 and I4 infections.

During fluoxetine treatment, the I227V mutation was observed in I3-F at week 4 o.t., and in both I3-F and I4-F at week 10 o.t. The A133T emerged in both infections at week 10 o.t. As for mutations R188G and A229V, they were only observed in I3-F infection at week 10 o.t.

Interestingly, these mutations were not observed in the virus obtained from infected cultures treated with DMSO (I1-D, I2-D, I3-D, and I4-D).

## 4. Discussion

The investigation of existing drugs with well-established safety profiles for new indications is a cheaper and faster strategy to discover new antiviral agents. Indeed, the screening of approved molecule libraries allowed to identify previously unrecognized inhibitors of enterovirus replication, including fluoxetine [11,15].

We have previously shown that fluoxetine can successfully clear persistent CVB4 E2 infection within a month when cultures were treated at 5.48 μM, twice a week [12]. Further experiments described in this study revealed a failure to clear the virus in some persistent CVB4 E2 infections despite a long-term fluoxetine treatment. The lack of susceptibility to fluoxetine in these “resistant” isolates was confirmed in a model of acute infection using HEp-2 cells.

Fluoxetine and other described enterovirus inhibitors were shown to exert antiviral activity by targeting 2C protein that is one of the most conserved and complex nonstructural viral proteins among picornaviruses [11,15]. This protein was reported to be involved in several key events throughout the virus life cycle (different steps of replication, immune evasion…), but its precise role is not fully understood. 2C harbors an N-terminal membrane-binding motif, an adenosine triphosphatase (ATPase) domain, a cysteine-rich motif, and RNA binding sites [16]. The ATPase domain, which belongs to SF3 helicases of the AAA+ ATPase superfamily, contains Walker motifs (motifs A and B) and motif C [17]. ATPase activity has been earlier demonstrated for 2C protein [8]. However, every attempt to determine the helicase activity associated with 2C ATPase has failed until recently when a study provided evidence of this helicase activity in the 2C protein of EV-A71 and CV-A16 [18].

One of the major challenges to overcome during the investigation or the development of an antiviral agent, especially a direct acting agent, is the emergence of resistant mutants. This is particularly true for RNA viruses that usually generate a significant number of mutations during the replication process due to a poor proofreading activity of RNA polymerase [19].

Our model of persistent CVB4 E2 infection in pancreatic cell cultures is attractive to investigate the resistance to antiviral molecules, because it allows multiple and long-term exposition of virus to molecules. This drug pressure promotes the emergence of resistant mutants, which more or less quickly results in failure of virus clearance, depending on the resistance barrier of the drug.

Previous studies have described three residues substitutions in 2C protein (A224V, I227V, A229V), that confer CVB3 resistance to fluoxetine, TBZE-029, GuHCl, and other recently identified 2C targeting inhibitors [8,15]. These substitutions are present in a short stretch of amino acids 224AGSINA229 located immediately at the C terminal of ATPase motif C. The AGSINA motif was found to be conserved in *Enterovirus B* (such as CVB4) and *D* species but is not present in other enteroviruses [10].

In this study, we found two of these substitutions in the fluoxetine-resistant viral suspensions (both I227V and A229V (double population mutant/wild type) in I3-F, and only I227V in I4-F). These changes were not observed before treatment, therefore, have probably emerged under drug-selection pressure. However, given the limit of Sanger sequencing, it cannot be excluded that these mutants preexisted as minor variants. The fluoxetine-resistant mutant used in reported studies (obtained by site-directed mutagenesis) harbored all these three mutations, and the impact of each mutation could not be clearly assessed [10,15]. In addition, the fact that, resistant viruses obtained from persistent infections treated with fluoxetine (I3-F and I4-F) were still susceptible to GuHCl, shows that the effect of these mutations (alone or combined) might depend on the 2C-inhibitor tested. Indeed, de Palma et al. [20]. previously reported a detailed impact of these mutations on the susceptibility to GuHCl. I227V alone did not induce resistance while the combination of I227V + A229V was associated with low-level resistance. Thus, the mutations observed in I3-F and I4-F seems to be insufficient to confer resistance to GuHCl in vitro.

In this report, other mutations not previously described were observed in the 2C ATPase domain of the resistant viruses (A133T and R188G in I3-F, and A133T in I4-F). I3-F, the viral suspension with the most mutations, appears to be the most resistant one to fluoxetine.

Interestingly, the R296G mutation was observed before treatment only in the virus obtained from persistent infections, which would later display resistance to fluoxetine treatment (I3 and I4). Even if viral suspensions from all of the untreated persistently infected cultures (I1, I2, I3, and I4) were susceptible to fluoxetine, the role of R296G mutation in the predisposition of resistance to fluoxetine cannot be excluded. This substitution is located in the zinc finger domain, downstream of the cysteine-rich motif which forms a zinc-binding site [21]. In addition, this residue seems to be conserved in *Enterovirus B* and *D* species (the EV species reported to be susceptible to fluoxetine), and variable in others species. We hypothesized that this mutation might favor other compensatory mutations including those associated with resistance to fluoxetine.

In this study, we focused on the 2C protein; however, the role of changes in other parts of the viral genome cannot be excluded. Site-directed mutagenesis studies are needed to precisely analyze the impact of these new substitutions on the susceptibility of CVB to fluoxetine and other 2C targeting enterovirus inhibitors.

Currently, fluoxetine is not available as a treatment for EV in humans. Nevertheless, the understanding of the inhibition mechanism and resistance profiles can be useful for the design of new compounds [10,22].

## 5. Conclusions

In conclusion, we took advantage of a model of persistent CVB4 E2 infection to describe the emergence of fluoxetine-resistant variants. In these variants, mutations in the 2C viral protein have been identified and deserve further investigation.

## Figures and Tables

**Table 1:** Primers used for sequencing.

Primer Name	Forward/Reverse	Primer Sequence (5′–3′)	Nucleotide Position
EXT-1	Forward	CTCAAGCGGAAAGTGTCCCA	3988–4007
INT-1	Reverse	TTTCCCATCAGGGTTCTGGC	4593–4574
INT-2	Forward	GATTGGGCGTTCACTTGCAG	4461–4480
EXT-2	Reverse	ACTGCCTCACTATCCACCGA	5126–5107

**Table 2:** Changes observed in 2C sequences.

2C Protein Sequences				Amino-Acid Positions						
				133	188	216	227	229	255	296
CVB4 E2 reference published strain (NCBI, accession: AF311939.1)				A	S	P	I	A	S	R
Laboratory CVB4 E2 stock strain				A	S	P	I	A	S	R
**Sequences of isolates from CVB4 E2 persistently infected Panc-1 cells**	Baseline samples (CVB4 E2 persistent infection, 20 weeks)		I1	A	S	P	I	A	N	R
			I2	A	S	S	I	A	S	R
			I3	A	S	P	I	A	S	G
			I4	A	S	P	I	A	S	G
	4 weeks post treatment	Fluoxetine	I1	ND	ND	ND	ND	ND	ND	ND
			I2	ND	ND	ND	ND	ND	ND	ND
			I3	A	S	P	I/V	A	S	G
			I4	A	S	P	I	A	S	G
		DMSO	I1	A	S	P	I	A	N	R
			I2	A	S	S	I	A	S	R
			I3	A	S	P	I	A	S	G
			I4	A	S	P	I	A	S	G
	10 weeks posttreatment	Fluoxetine	I1	ND	ND	ND	ND	ND	ND	ND
			I2	ND	ND	ND	ND	ND	ND	ND
			I3	T	S/A	P	V	A/V	S	G
			I4	A/T	S	P	V	A	S	G
		DMSO	I1	A	S	P	I	A	N	R
			I2	A	S	S	I	A	S	R
			I3	A	S	P	I	A	S	G
			I4	A	S	P	I	A	S	G

ND: Not done, virus undetectable; I3 and I4 are resistant to fluoxetine treatment. AA changes are underlined.
